# The effects of modifying mental imagery in adolescent social anxiety

**DOI:** 10.1371/journal.pone.0230826

**Published:** 2020-04-06

**Authors:** Eleanor Leigh, Kenny Chiu, David M. Clark

**Affiliations:** 1 Department of Experimental Psychology, University of Oxford, Oxford, United Kingdom; 2 Department of Psychology, Institute of Psychiatry, Psychology and Neuroscience, King’s College London, London, United Kingdom; Stanford University, UNITED STATES

## Abstract

**Background:**

The identification of negative self-imagery as a maintenance factor in adult social anxiety has led to enhanced treatments for this population. Whilst intrusive negative imagery is commonly reported by socially anxious adolescents, no studies have demonstrated that it plays a causal role in maintaining symptoms. To assess this possibility, we undertook an experimental study manipulating social self-imagery in high socially anxious adolescents.

**Methods:**

High socially anxious adolescents undertook two conversations under different conditions. During one conversation they held a negative social self-image in mind, and in the other they held a benign social self-image in mind. Self-report, conversation partner report and independent assessor ratings were taken.

**Results:**

When participants held a negative self-image in mind, they reported feeling more anxious, and believed they looked more anxious and performed more poorly. Furthermore, they overestimated how anxious they looked compared to conversation partner ratings. As well as distorting participants’ perception of their anxious appearance, holding a negative image in mind also had observable effects on the interaction. Participants were rated as looking more anxious and performing less well by their conversation partner when they held such images in mind, and the conversation was rated more critically by conversation partners and independent observers. Finally, a preliminary mediation analysis suggested that the detrimental effect of negative imagery on the social interaction may be partly due to the spontaneous use of avoidant safety behaviours.

**Conclusions:**

The findings provide support for a causal role of negative self-imagery in adolescent social anxiety and point to the potential clinical value of techniques targeting imagery to treat the disorder.

## Introduction

The majority of adults with social anxiety disorder (SAD) report experiencing intrusive negative and distorted images of themselves in social situations [1]. The images are usually closely linked to the individual’s particular fears, for example, a patient with a fear of shaking may notice a slight tremble in her hand which would trigger a distorted image of her hand shaking violently. Spontaneously occurring images are experienced from an observer’s perspective [1], and so it is understandable that patients assume the images are accurate. Clark and Wells [2] suggested that these excessively negative images are causally implicated in the maintenance of social anxiety. Specifically, the experience of negative images increases anxiety and motivates the use of unhelpful safety behaviours. Safety behaviours are intended to prevent or minimise a feared outcome from occurring, and may involve avoidance (e.g. avoiding eye contact, speaking less), or management of the impression one conveys (e.g. preparing topics of conversation in advance, monitoring) [3]. It has been suggested that avoidance safety behaviours may be especially unhelpful as they convey a disinterested or unfriendly impression, thereby ‘contaminating’ the social interaction.

Experimental studies that have directly manipulated self-imagery in socially anxious adults are consistent with the assertion that negative images maintain social anxiety in adults. For example, in a pair of studies, Hirsch et al. [4, 5] experimentally manipulated imagery amongst high socially anxious adults during a conversation task. They found that holding a negative (compared to a neutral) image in mind made participants feel more anxious, believe that they performed less well, increased the spontaneous use of safety behaviours, and led to more negative appraisals by others. Furthermore, an association was found between the use of avoidant safety behaviours and more critical ratings by the conversation partner, but not with impression management safety behaviours, consistent with the notion that avoidant safety behaviours are particularly detrimental. These findings have paved the way for the development of specific therapeutic techniques aimed at correcting distorted self-imagery, most notably video feedback [6].

The question of whether intrusive negative self-imagery is also relevant to adolescent social anxiety has been addressed only partially up to now. It would seem to be a pertinent question given the increase in social worries [7] and social anxiety disorder [8] during the adolescent period. Furthermore, it is possible that intrusive negative images are more problematic in adolescence, when individuals are less able to attain cognitive control over mental imagery [9]. Three observational studies have reported on the relationship between social anxiety and negative self-imagery in adolescents and a medium association was reported [10–12], for a review see Leigh and Clark [13]. However, all three studies were correlational and cross-sectional and so cannot answer the question of whether negative self-imagery plays a causal role in maintaining adolescent social anxiety. Experimental studies, in which a process of interest is manipulated and the effects measured, provide a powerful method of testing causation. The only experimental study that has manipulated self-imagery in adolescents was undertaken by Alfano and colleagues [14]. Adolescents with SAD and healthy controls took part in videotaped role-play and read-aloud tasks and then rated their anxiety and performance. An independent assessor also rated their performance. Half of the control participants were instructed to engage in negative self-imagery during the tasks. The other half of the control group and the SAD group received no instructions for the tasks. The SAD group was rated as more anxious and less competent, whilst minimal differences were found between the control groups. Whilst this could be because negative self-imagery is a consequence rather than a cause of social anxiety, design issues could also explain the findings. In particular, for the control group with low symptoms of social anxiety, the experience of negative self-imagery in social settings will be relatively rare, and so it is likely that they would have found it difficult to generate and maintain negative self-images. In our study we addressed this issue by selecting a group of adolescents with high social anxiety, then experimentally manipulating spontaneously occurring self-imagery. This provides a more robust test of the hypothesis that negative self-imagery is causally implicated in adolescent social anxiety. If in this population we also fail to observe differences as seen in the adult population then it would suggest that the mechanisms underlying social anxiety may be different in adolescents and adults. In the present study, we examined the perceived and actual effects of negative self-imagery compared to benign self-imagery during a conversation task using a within-subjects design. Three sources of outcome data were compared: self-report, conversation partner-report, and independent assessor ratings.

## Recruitment

### Ethical approvals and consent/assent procedures

The study received ethical approval from the University of Oxford Medical Sciences Division Ethics Committee (CUREC Reference: R54283/RE001). Written parental consent and written young person assent were obtained for participation in the study.

### Sample size estimation

Power analysis was conducted *a priori* with reference to two studies that used similar experimental designs, one with a sample of socially anxious undergraduates and one with a clinical adult sample (Hirsch et al., 2003; 2004). The calculation estimated that in order to detect a difference between the imagery conditions with p < .05 and 80% power, 30 participants or more were required to test the probability of rejecting the null hypothesis when it is false. We increased the sample size to n = 34 given the differences in population.

### Recruitment procedure

Participants were recruited via a screening program undertaken in two secondary schools. All pupils in UK school years 7–9 (aged 11–14 years) were invited to take part. As part of the screening they completed a measure of social anxiety (the self-report version of the Liebowitz Social Anxiety Scale for Children and Adolescents, LSAS-CA [15]). Pupils who scored in the top quartile of the distribution of scores for their year group on the LSAS-CA were invited to take part. The LSAS-CA was repeated at the experimental testing session, and any participant whose score had changed such that they were no longer scoring within the upper quartile for their year group were not included (*n* = 0).

## Methods

### Design

High socially anxious participants had two conversations with a naïve conversation partner (gender-matched with participants). In one condition they held a negative self-image in mind and in the other they held a benign self-image in mind. The order of imagery condition was counterbalanced across participants. After each conversation, participants rated their anxiety and how well they thought they performed, and completed manipulation checks. They also completed questionnaires assessing safety behaviour use and observable aspects of behaviour. Conversation partners rated participants’ performance, the conversation and their own anxiety after each conversation. Participants and conversation partners also both completed a questionnaire assessing the quality of the conversation. The conversations were videotaped and an independent assessor blind to the study aims and experimental manipulation rated the interaction on the conversation quality questionnaire.

### Participants

#### Participants

34 young people, 22 of whom were female, served as the experimental participants. Their average age was 13.55 (*SD* = 0.88), ranging from 11.85 to 14.79 years. The average total score on the LSAS-CA was 71.03 (*SD* = 27.57), ranging from 21 to 116. The mean score on the Short Mood and Feelings Questionnaire (18) was 9.91 (*SD* = 5.58), ranging from 2 to 22.

#### Conversation partners

Six psychology students (50% female) served as the conversation partners. It was explained to them that they were helping with a psychology study about adolescents, but they were naïve to the specific study aims and manipulation. Their average age was 22 years (*SD* = 3.36; range = 16–24), and the average LSAS score was 26 (*SD* = 12.01; range = 11–39).

### Manipulation of imagery

Each participant generated two images: One that they typically experienced in socially anxious situations (the ‘negative’ image) and the other that they experienced when they felt relaxed or less anxious (the ‘benign’ image). The procedure to generate the images was the same as that reported by Hirsch et al. [4, 5], based on Hackmann et al. [1]. Minor adaptations were made and piloted to ensure the instructions were appropriate for adolescents. The procedure involved two stages; a situation was identified and then the associated image was elicited. For the negative image condition, participants recalled a social situation when they had felt anxious, and for the benign image they recalled a social situation when they had felt relaxed. Once the situation had been identified, the associated image was elicited by asking participants a series of questions about how they felt they looked and sounded, felt, and how they came across to others in the situation.

### Materials

*Social anxiety symptoms* were measured with the self-report version of the 24 item Liebowitz Social Anxiety Scale for Children and Adolescents, LSAS-CA [15], which ranges from 0 to 144 (with higher scores indicating more social anxiety symptoms). Psychometric properties of the scale are sound [16] and internal consistency in the current study was high (Cronbach’s α = .95).

*Depression symptoms* were measured with the short form of the Mood and Feelings Questionnaire (SMFQ [17]). Internal consistency in the present sample was high (Cronbach’s α = .86).

Three visual analogue scale (VAS) ratings were included as *Manipulation Checks*. Participants were asked to rate: how able they were to hold the image in mind during the conversation (range = 0–100); the clarity of the image (range = 0–100); and the valence of the image (from -3, totally negative, to +3, totally positive).

*Anxiety and Performance Ratings* were made after each conversation on VAS. Participants were asked to rate how anxious they felt and how anxious they thought they appeared (from 0 ‘not at all anxious’ to 100 ‘very severely anxious’). Lastly, they were asked how well the conversation went overall (from 0 ‘not at all well’ to 100 ‘really well’).

Sixteen items assessing safety behaviour use were taken from the *Adolescent Social Behaviour Questionnaire (ASBQ*; each item is scored on a 4-point scale ranging from 0–3, adapted from the adult version [18]). Participants completed these 16 items twice during the study, once after each conversation. They were asked to make their ratings based on what they had done during the preceding conversation only. Internal consistency was high (Cronbach’s α = .93). In line with Hirsch et al. [4], items on the ASBQ were divided into two categories, ‘Avoidance’ (10 items; Cronbach’s α = .89) and ‘Impression Management’ (6 items; Cronbach’s α = .89).

The self-report version of the *Behaviour Questionnaire* developed by Hirsch et al. [4] was completed after each conversation. The questionnaire measures observable aspects of behaviour, with higher scores indicating more observable anxiety and poorer performance. Total scores (Cronbach’s α = .91) and subscales for specific negative (Cronbach’s α = .81), global negative behaviours (Cronbach’s α = .83) and positive behaviours (Cronbach’s α = .83) were calculated.

*Conversation Partner Ratings* were obtained after each conversation on the following scales: how anxious the participant appeared (from 0 ‘not at all anxious’ to 100 ‘very anxious’); how enjoyable the conversation was (from 0 ‘not at all enjoyable’ to 100 ‘extremely enjoyable’); how likeable they found the participant (-50 ‘less likeable than average’ to +50 ‘more likeable than average’); and the conversation partner’s own anxiety (from 0 ‘not at all anxious’ to 100 ‘very anxious’).

The 12-item *Conversation Questionnaire* was completed by both participant and conversation partner after each conversation. It was developed by Hirsch et al [4] and assesses various aspects of the conversation, such as flow, pauses, and reciprocity. Higher scores indicate a more critical evaluation of the conversation (Self-report Cronbach’s α = .86; Conversation partner Cronbach’s α = .88; Independent rater Cronbach’s α = .94). An independent rater (psychology graduate) also scored the conversations on the *Conversation Questionnaire*, modified for observer rating (e.g. *‘I interrupted the conversation partner’* changed to ‘*The participant interrupted the conversation partner’*). The rater demonstrated excellent interrater reliability on a subsample of 50% of the videos rated by the lead author (*r* = .95), and the internal consistency of the scale was high (α = .95).

### Procedure

Testing took place in school during school hours. Before beginning, it was explained to conversation partners that they would be taking part in brief conversations with young people. They were asked to imagine that they were meeting them for the first time at a family party or social event. They were instructed not to talk about the experiment during the conversation. Participants initially completed the LSAS-CA and the SMFQ. They were allocated to ‘Negative—Benign’ or ‘Benign—Negative’ imagery conditions in counterbalanced order. They then underwent the manipulation (see *Manipulation of imagery* for details). Participants were asked to continue following the instructions during the conversation with the conversation partner whom they had not met before. Participants were given a conversation topic (either ‘Entertainment’ or ‘Holidays’, these were assigned in counterbalanced order within conditions). The conversation partner was unaware of the study design. The conversation partner was also given the conversation topic. The participant and stooge were introduced to one another and the experimenter sat discreetly in the room during the conversation. Each conversation lasted 5 minutes. After each conversation, participants completed *Manipulation Checks*, *Anxiety and Performance Ratings*, *ASBQ Items*, *Behaviour Questionnaire*, and *Conversation Questionnaire*. The conversation partner completed *Conversation Partner Ratings* and *Conversation Questionnaire*. At the end of the session participants were thanked for their time and debriefed.

### Data analysis

To compare the effects of negative and benign imagery on self-report ratings, MANOVA were undertaken, with one within-subjects factor, (Condition (Negative and Benign)) and one between-subjects factor (Order (Negative-Benign and Benign- Negative)).

To examine the extent to which participants overestimated how anxious they looked, we calculated a difference score (self-reported anxious appearance minus conversation partner reported anxious appearance) in the negative and benign imagery conditions, and compared these in an ANOVA.

To examine whether there were observable effects of negative compared to benign imagery, MANOVA were undertaken on conversation partner ratings, with one within-subjects factor, (Condition (Negative and Benign)) and one between-subjects factor (Order (Negative-Benign and Benign- Negative)). To examine the effects of negative and benign imagery on Conversation Questionnaire ratings, a 3-way ANOVA was run, which included Rater as a within-subjects factor (Participant, Conversation Partner, Observer), as well as Condition and Order factors.

All MANOVA and ANOVA were run with Topic Order as a further between subjects variable (Holidays–Entertainment vs. Entertainment–Holidays). No main effects or interactions were significant and therefore analyses are reported without the inclusion of this factor.

To examine the hypothesis that the detrimental effect of negative imagery on participants’ social performance is due to avoidant safety behaviours, we undertook mediation analyses of the effect of negative (vs. benign) imagery on conversation partner-rated and observer-rated conversation quality, with avoidant and impression management safety behaviours as separate mediators.

### Results

#### Manipulation checks

Table 1 summarises means and standard deviations, and test statistics. MANOVA revealed no significant main effects or interactions for clarity of the image, or ability to hold the image in mind, suggesting participants rated themselves as able to comply with instructions equally well in both conditions. As expected, there was a main effect of condition on the valence of the image, with more negative images reported in the negative imagery condition. No other main effects or interactions were significant.

**Table 1:** Means and standard deviations for outcome variables in the negative and the benign imagery conditions, with test statistic and estimate of the effect size for this contrast (Multivariate tests revealed the only significant effect was Condition, therefore results of the univariate tests for this contrast only are presented).

Measure	Condition		Test Statistic	*p* value	Partial eta^2^ (η_p_^2^)
	Negative Imagery Mean [SD]	Neutral Imagery Mean [SD]			
*Manipulation Checks*					
Held image in mind (0–100)	69.71 [21.67]	73.24 [19.61]	*F*(1, 32) = 0.53	.47	0.02
Valence (-3 - +3)	-1.60 [0.95]	2.09 [0.61]	*F*(1, 32) = 292.89	< .001	0.90
Clarity (0–100)	60.44 [24.07]	59.41 [22.01]	*F*(1, 32) = 0.04	.85	0.00
*Self-Report Anxiety and Performance Ratings*					
Feel anxious (0–100)	65.44 [16.44]	26.32 [16.98]	*F*(1, 32) = 155.94	< .01	0.83
Look anxious (0–100)	70.15 [18.57]	32.65 [20.24]	*F*(1, 32) = 102.73	< .01	0.76
How well the conversation went (0–100)	48.53 [21.59]	76.24 [18.08]	*F*(1, 32) = 57.37	< .01	0.64
*Overestimation of Anxious Appearance*	16.03 [32.14]	-1.03 [27.57]	F(1, 32) = 15.10	< .01	0.32
*Behaviour Questionnaire*					
Total Score	65.06 [18.22]	31.44 [16.05]			
Global Negative Behaviour	28.32 [7.79]	13.56 [7.31]	*F*(1, 32) = 155.32	< .01	0.83
Specific Negative Behaviour	14.53 [6.97]	6.97 [5.84]	*F*(1, 32) = 68.45	< .01	0.68
Positive Behaviour	22.21 [7.26]	10.91 [5.77]	*F*(1, 32) = 104.97	< .01	0.77
*Safety Behaviour Questionnaire*					
Total	69.24 [22.67]	45.71 [21.59]	*F*(1, 32) = 105.01	< .01	0.77
Avoidance Behaviours	41.50 [15.23]	24.12 [13.71]	*F*(1, 32) = 98.18	< .01	0.75
Impression Management	27.74 [9.56]	21.59 [9.36]	*F*(1, 32) = 36.02	< .01	0.53
*Conversation Partner Ratings*					
Enjoyed conversation (0–100)	43.24 [22.22]	63.68 [20.35]	*F*(1, 32) = 26.04	< .01	0.45
Participant looked anxious (0–100)	54.12 [26.04]	33.68 [24.00]	*F*(1, 32) = 28.31	< .01	0.47
Liked the participant (-50 - +50)	2.50 [18.84]	15.88 [19.36]	*F*(1, 32) = 29.17	< .01	0.48
Own feelings of anxiety (0–100)	27.50 [23.72]	20.00 [18.22]	*F*(1, 32) = 8.50	< .01	0.21
Wanted to continue conversation (0–100)	40.88 [22.34]	62.06 [23.71]	*F*(1, 32) = 28.37	< .01	0.47
*Conversation Questionnaire Ratings*					
Participant Rater	45.19 [13.10]	26.90 [11.22]			
Conversation Partner Rater	45.26 [13.52]	32.90 [15.99]	see text		
Independent Rater	34.32 [20.43]	21.84 [14.39]			

#### Self-report ratings

Means and standard deviations are shown in Table 1. There was a main effect of condition across all Anxiety and Performance Ratings, with participants rating themselves as feeling and looking less anxious, and coming across better in the benign imagery condition. There was a significant main effect of condition on the Behaviour Questionnaire, with more critical ratings in the negative imagery condition, for the total and subtotal scores. Participants reported using significantly more safety behaviours (total and subtypes) in the negative imagery condition. No other effects were significant.

#### Overestimation of anxious appearance

ANOVA revealed a significant main effect of condition, with participants overestimating how anxious they appeared more when holding a negative image in mind compared to a benign image (see Table 1). No other main effects or interactions were significant.

#### Conversation partner ratings

There was a significant main effect of condition on all Conversation Partner Ratings, see Table 1, with more critical ratings in the negative compared to the benign condition. No other effects were significant.

#### Conversation Quality Questionnaire (Self, Conversation Partner, and Observer Rated)

Three-way ANOVA revealed a main effect of condition (*F*(1, 32) = 41.69, *p* < .01, η_p_^2^ = .57), with the conversation rated more negatively for the negative compared to the benign imagery condition by participants (see Table 1 for means and standard deviations). No other effects were significant.

#### Mediation analysis

Mediation analyses were undertaken to examine the effect of negative (vs. benign) imagery (Y) on conversation partner-rated and observer-rated conversation quality (X), with avoidant and impression management safety behaviours as separate mediators (M). The direct, indirect, and total effects were estimated using the MEMORE macro for SPSS [19]. The magnitude and 95% confidence intervals of the indirect effects were estimated (using 5000 bootstrap resamples, with replacement). Significance of the indirect effect was indicated by the 95% confidence interval not crossing zero.

In line with the hypotheses, the indirect (mediation) effect for the Avoidance subscale of the ASBQ was significant (bootstrap coefficient = 10.58, *SE* = 4.13), with a 95% confidence interval ranged from 2.52 to 18.91, for the conversation partner rated conversation questionnaire, and also for the observer rated conversation questionnaire (bootstrap coefficient = 1.02, *SE* = 0.46, [95% CI: 0.05, 1.88]). Also in line with hypotheses, the indirect (mediation) effect for the Impression Management subscale of the ASBQ was not significant for either the partner (bootstrap coefficient = 1.27, *SE* = 2.43, [95% CI: -2.93, 6.90]), or the observer rated conversation questionnaire (bootstrap coefficient = 0.22, *SE* = 0.30, [95% CI: -0.26, 0.90]).

## Discussion

Identifying cognitive processes that maintain adolescent social anxiety and that are modifiable is important because it provides a route to optimising treatments. Negative self-imagery, that we know is causally implicated in adult social anxiety, is one such candidate process. However, whilst previous research with adolescents has demonstrated that negative self-imagery is associated with social anxiety, the only experimental study undertaken in adolescents reported no effect of manipulating imagery in healthy volunteers [14]. We were interested to examine whether this is because negative self-imagery operates differently in adolescents compared to adults, or whether the null finding is related to design issues. Therefore, in the present study we experimentally manipulated self-imagery in high socially anxious adolescents. We found that holding a negative image in mind did have a detrimental effect across self-report, conversation partner, and independent assessor ratings. The findings provide the first direct evidence that negative self-imagery is causally implicated in social anxiety in adolescents as it is in adults, rather than an epiphenomenon or consequence.

High socially anxious adolescents reported feeling more anxious when holding a negative self-image in mind compared to a benign image. They also thought that they came across less well and that the conversation was less successful, for example, that there were more pauses and it flowed less well. Participants thought that they looked more anxious, with higher ratings of observable aspects of anxiety, such as fidgeting and blushing when holding a negative image in mind.

Our findings suggest that the more critical self-judgements associated with holding a negative compared to a benign image in mind are a result of distorted perception and also partly based in reality. The extent to which participants have a distorted perception of how they come across can be measured by comparing self and conversation partner ratings of anxious appearance. What we find is that when adolescents hold a negative image in mind, they overestimate how anxious they look. This is in contrast to the fairly accurate assessment they make of their anxious appearance when holding a benign image in mind. The present data suggests that the use of internal information, such as negative self-imagery, is associated with this distortion when holding a negative image in mind. It raises the question of whether socially anxious adolescents use other internal information to infer how they come across, in addition to self-imagery. For example, there is evidence to suggest that socially anxious adults use physical sensations of anxiety to (over) estimate how anxious they look [20]. This hypothesis has not yet been tested in adolescents.

There is also some reality to participants’ critical self-judgements. Conversation partners and the independent observer, naïve to the study design, also rated the conversation more critically when the participant held a negative image in mind. They rated the conversation as less reciprocal, interesting and enjoyable, and conversation partners rated the participant as looking more anxious. As images are internal events, it may seem surprising that they influence the way our behaviour is rated by other people. How could self-imagery affect observable behaviour? It could be that participants felt much more anxious in the negative image condition and the visible effects of anxiety affected the whole interaction. Another possibility is that it is due to the contaminating effect of safety behaviours, and avoidant safety behaviours in particular. Indeed, in the negative imagery condition, participants reported using more safety behaviours in general compared to the benign imagery condition, despite no instructions to do so, and so it seems that negative self-imagery motivates the spontaneous use of safety behaviours in socially anxious adolescents.

It may be that the increase in safety behaviour use, and avoidant safety behaviours in particular, impacts on how other people experience the conversation. Our preliminary mediation analysis suggests that this may be the case: avoidant but not impression management safety behaviours mediated the relationship between negative imagery and both conversation partner and observer ratings of the conversation quality. By way of example, a socially anxious adolescent who stands on the edge of the group, avoiding eye contact and declining to ask any questions for fear of stumbling over their words and appearing stupid, will be perceived more critically by others. This is a sad irony, because this is the opposite message that they are trying to communicate. However, it is important to note that our sample size was substantially lower than what is considered optimal for mediation analysis [21]. Therefore this finding could be a false positive and replication with a larger sample size is needed. We have previously suggested that adolescents may rely more heavily on avoidant safety behaviours rather than impression management strategies, compared to adults [22]. If this were borne out in future studies, then it would suggest that socially anxious adolescents are particularly vulnerable to becoming caught in vicious cycles of anxiety, safety behaviour use, and peer rejection.

Importantly, the manipulation used in the study appears to be robust. Participants were able to hold a clear and vivid image in mind throughout the task, with no differences between conditions except for valence of the image, as intended. These ratings suggest that adolescents (at least those aged 11–14 years), like adults, are able to generate an image of themselves and hold this in mind, but also that the valence can be explicitly manipulated. Extending beyond this study, future research examining if and how the relationship between imagery and social anxiety changes during the course of childhood and adolescence will be important, given that competence in mental imagery (i.e. in the ability to generate, inspect, maintain and manipulate images [23]), is likely to develop over the course of this period [9].

The study is not without its limitations. Whilst the study was adequately powered, the sample size was modest. In addition, we note that conversation partners were on average nine years older than participants, which may limit generalisability to an extent, and future studies with similar-aged peers as conversation partners would be valuable. For example, it may be that other adolescents, who as a group are typically sensitive to social reward and rejection, may be more affected by social anxiety- related avoidant behaviours than adults. Furthermore, we note that baseline ratings of anxiety and related self-report measures were not gathered. It would be interesting to chart whether changes from baseline across time may have differed between conditions.

The present study has yielded consistent findings pointing to the detrimental effect of negative imagery in social anxiety for adolescents. Although the sample was not drawn from a clinical population, participants’ average score on a measure of social anxiety (LSAS-CA) was high (*M* = 71.03 [*SD* = 27.57]) and the participant with the lowest LSAS-CA scored 21 (and the next lowest LSAS-CA score was 30), which is only marginally below the suggested threshold (22.5) for distinguishing adolescents with social phobia and healthy controls [16]. Taken together, this suggests that the findings from this study can be informative about clinical interventions for socially anxious adolescents. Techniques to target negative imagery may be beneficial, for example the use of video feedback to correct distorted images is a core technique in Cognitive Therapy for social anxiety in adults, e.g. [24], and shows promise for adolescents [25, 26]. Specific support for the use of this technique with adolescents came from a study by Parr and Cartwright-Hatton [27]. Compared to a control condition, they demonstrated that video feedback with careful verbal preparation (see also [6]) led to reductions in anxiety and more positive predictions amongst socially anxious teenagers.

## Abbreviations

SA: Social Anxiety
SAD: Social Anxiety Disorder
VAS: Visual Analogue Scales
